# Relationship among Low T3 Levels, Type 3 Deiodinase, Oxidative Stress, and Mortality in Sepsis and Septic Shock: Defining Patient Outcomes

**DOI:** 10.3390/ijms24043935

**Published:** 2023-02-15

**Authors:** Josi Vidart, Luiza Axelrud, André Cardoso Braun, Rafael Aguiar Marschner, Simone Magagnin Wajner

**Affiliations:** 1 Thyroid Section, Endocrine Division, Hospital de Clínicas de Porto Alegre, Universidade Federal do Rio Grande do Sul, Porto Alegre 90035-003, Rio Grande do Sul, Brazil; 2 Department of Internal Medicine, Universidade Federal do Rio Grande do Sul, Porto Alegre 90035-003, Rio Grande do Sul, Brazil

**Keywords:** low T3 syndrome, oxidative stress, sepsis, septic shock, thyroid hormone, type 3 deiodinase

## Abstract

Low T3 syndrome occurs frequently in patients with sepsis. Type 3 deiodinase (DIO3) is present in immune cells, but there is no description of its presence in patients with sepsis. Here, we aimed to determine the prognostic impact of thyroid hormones levels (TH), measured on ICU admission, on mortality and evolution to chronic critical illness (CCI) and the presence of DIO3 in white cells. We used a prospective cohort study with a follow-up for 28 days or deceased. Low T3 levels at admission were present in 86.5% of the patients. DIO3 was induced by 55% of blood immune cells. The cutoff value of 60 pg/mL for T3 displayed a sensitivity of 81% and specificity of 64% for predicting death, with an odds ratio of 4.89. Lower T3 yielded an area under the receiver operating characteristic curve of 0.76 for mortality and 0.75 for evolution to CCI, thus displaying better performance than commonly used prognostic scores. The high expression of DIO3 in white cells provides a novel mechanism to explain the reduction in T3 levels in sepsis patients. Further, low T3 levels independently predict progression to CCI and mortality within 28 days for sepsis and septic shock patients.

## 1. Introduction

Non-thyroidal illness syndrome (NTIS) refers to characteristic changes in thyroid hormone (TH) levels in response to systemic illness. Typical changes include low plasma concentrations of triiodothyronine (T3), low or normal thyroxine (T4), and elevated reverse T3 (rT3) in the presence of inadequate normal levels of thyrotropin (TSH), pointing to a loss of the neuroendocrine feedback mechanism [1,2]. It is known that THs are regulated by the activity of types 1 (DIO1), 2 (DIO2), and 3 (DIO3) deiodinases. DIO1 and DIO2 convert T4 to T3, the active hormone [3]. DIO3 is the most important inactivating enzyme and converts T4 and T3 into rT3 and other inactive forms [4]. Low serum T3 levels are associated with increased mortality [5,6,7,8,9,10], prolonged weaning from mechanical ventilation [11,12], and reduced cardiac output [13]; however, some studies failed to demonstrate any prognostic implications [14,15,16].

Sepsis is one of the most common indications for intensive care unit (ICU) admission and a major cause of morbidity and mortality worldwide [17]. A considerable number of patients still endure prolonged, complicated ICU stays, thus becoming chronically and critically ill. The term “chronic critical illness” (CCI) describes patients with ICU admission >7 days and who require complex medical care and become dependent on one or more life support systems, leading to functional dependence and poor long-term survival [18,19]. High levels of interleukin-6 (IL-6) is one of the hallmarks of sepsis and augments the production of reactive oxygen species (ROS), compromising deiodinase function and altering the peripheral T3/T4 activation/inactivation process [20]. N-acetylcysteine (NAC), an antioxidant that replenishes glutathione (GSH) and thiols, counteracts the effects of IL-6 on deiodinase-mediated T4 to T3 conversion. This indicates that IL-6 inhibits the function of deiodinases by increasing cellular ROS, which subsequently reduces the levels of GSH or GSH-like endogenous cofactors [21]. Among critically ill patients, those with sepsis present with pronounced increases in IL-6 production, compared to other critically ill patients with alternate diagnoses of similar severity [22]. Despite the ability to measure deiodinase function in deceased patients, measurement in living patients is limited since it requires biopsy samples.

We have seen that, despite the localization of sepsis to the initial site of disease, oxidative stress occurs in multiple tissues and results in the systemic induction of DIO3, suggesting that normal circulating thyroid hormone levels are dependent on the maintenance of a reduced cellular redox environment throughout the whole body [23]. Additionally, TH levels may predict mortality in hospitalized patients, in different clinical settings [24,25,26,27]. Moreover, it is also known that DIO3 is present in neutrophils [28] and in mice on macrophages of injured muscle (27). DIO3′s role in humans during sepsis or septic shock has not been addressed yet. In this novel study, we aimed to measure DIO3 expression in blood obtained from patients with sepsis and septic shock and determine its association with low T3 and the impact on mortality and the evolution to CCI. Therefore, we determined the relationship between T3 levels during sepsis stages and the levels of deiodinase expression in vivo.

## 2. Results

### 2.1. Study Population and TH Levels at ICU Admission

Of the 260 patients, 66.1% presented with septic shock (Table 1). Pneumonia was the most common cause of sepsis (47%). The mean age was 58.3 ± 14 years, and 56% of the patients were male. There were significant differences in the severity scores between survivors and non-survivors. Low T3 on admission was present in 225 patients (86.5%). Of these, 100 patients (38.4%) had both T3 and T4 levels below the reference range. The T3 mean values were below the normal range in all patients, with a statistically significant difference between those with sepsis and septic shock (*p* < 0.001). The TSH, T4, and free T4 (fT4) mean values were within the normal range in all groups. The mortality was 14.8% and 47.1% in patients with sepsis and septic shock, respectively.

**Figure 1:**
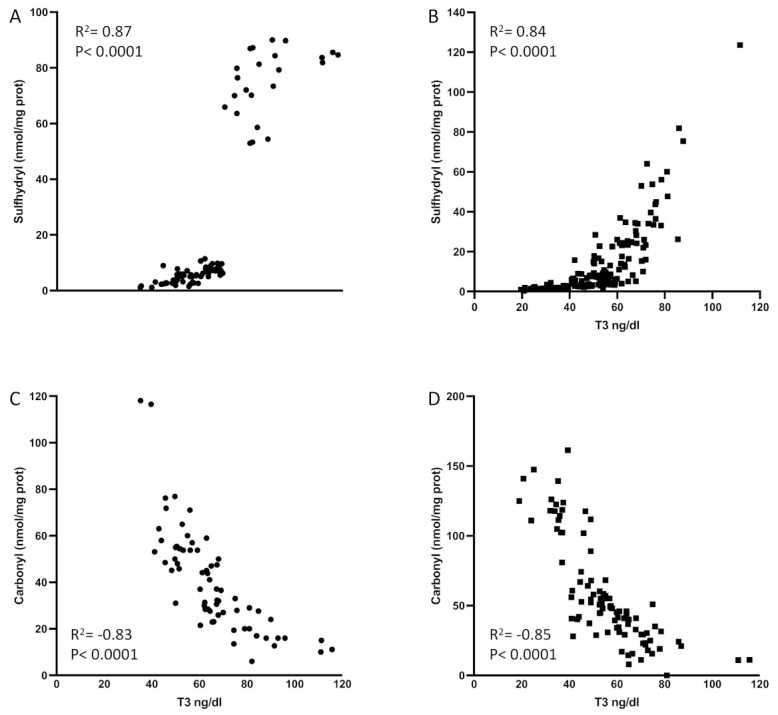
Sulfhydryl levels are correlated with serum T3 in patients with sepsis (**A**) and septic shock (**B**). Carbonyl levels are inversely correlated with T3 levels in patients with sepsis (**C**) and septic shock (**D**). No outliers were omitted in this analysis. The *p*-levels are denoted in the graphs. T3: triiodothyronine. Spearman test was used to perform statistics.

The levels of T3 were strongly correlated with sulfhydryl levels or the total amount of circulating thiol groups (Figure 1). The levels of sulfhydryl were lower in septic shock patients, mirroring their low levels of T3 (R^2^ = 0.72; *p* < 0.0001, Figure 1B).

Interestingly, we observed an inverse correlation between carbonyl levels (oxidized proteins) and T3 levels in septic patients (R^2^ = 0.59, *p* < 0.0001; Figure 1C) as well as in septic shock (R^2^ = 0.66, *p* < 0.0001; Figure 1D). These results thus confirmed the importance of circulating reducing thiol groups to maintain normal levels of GSH and T3 in disease, as shown before [21]. Figure 1 displays patients with sepsis or septic shock and low T3 syndrome (T3 lower than 60 ng/dL) and patients with sepsis or septic shock and a higher or normal levels of T3, all those that composed the cohort of ICU patients described in this manuscript. This explains the two clusters we might have in the graphs.

### 2.2. DIO3 Is Substantially Induced in the Immune Cells of Patients with Sepsis and Septic Shock and Correlates with Mortality

**Figure 2:**
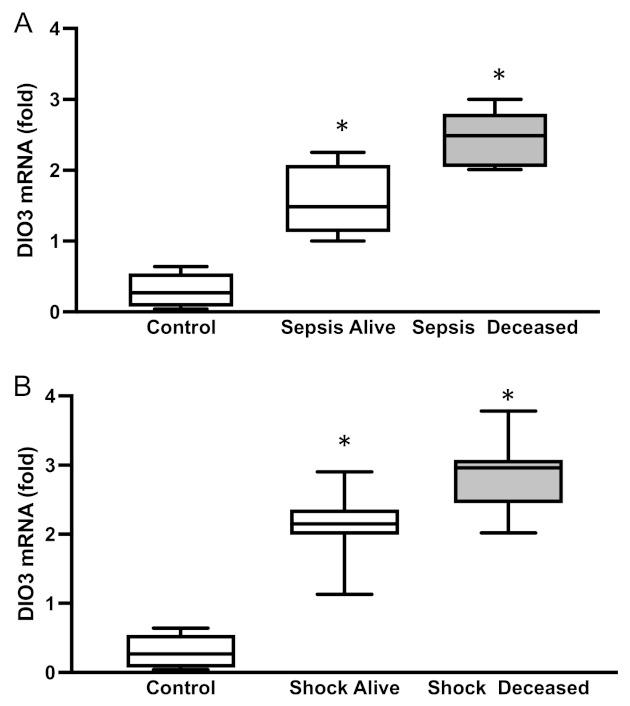
*DIO3* mRNA levels in patients with (**A**) sepsis or (**B**) septic shock. Mean ± SEM, * *p* < 0.0001 by ANOVA. D3: type 3 deiodinase.

DIO3 expression was very low in the immune cells of the healthy control group. However, we detected a 55% increase in DIO3 expression in patients with sepsis (*p* = 0.0002, Figure 2A). In patients with septic shock, DIO3 expression was higher than in the sepsis group. Interestingly, DIO3 expression was further increased (34% higher) in the deceased patients (*p* = 0.005, Figure 2B). When comparing survivors in both groups, we did not observe any increase in DIO3 expression.

### 2.3. T3 Levels at ICU Admission Are an Independent Predictor of 28-Day Mortality and Evolution to CCI

**Figure 3:**
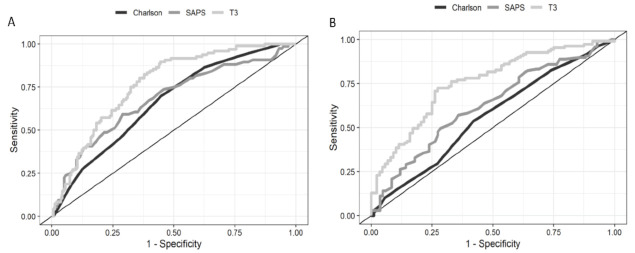
Receiver operating characteristic curves of T3 to predict 28-day mortality. (**A**) The AUC for lower levels of T3 is 0.76 (95% CI, 0.70–0.82). (**B**) ROC curves of T3 to predict the evolution to CCI. The AUC for lower levels of T3 is 0.75 (95% CI, 0.68–0.82). T3: triiodothyronine; ROC: receiver operating characteristic; and AUC: area under the ROC curve.

Patients who presented with NTIS at ICU admission revealed higher mortality than those with normal TH levels (41.3% *p* < 0.001, Table 1). Patients with low T3 and T4 levels had worse outcomes compared with those with only low T3 levels (mortality of 55% vs. 30%, *p* < 0.001). There were significant differences between survivors and non-survivors in the univariate analysis for the following variables: age; serum lactate levels; C-reactive protein levels; T3, T4, and fT4 levels; SAPS III; SOFA score; and Charlson Comorbidity Index. Lower levels of T3 yielded an independent AUC of 0.76 (Figure 3A). The AUC for SAPS III and the Charlson Comorbidity Index was 0.68 (95% CI, 0.60–0.76) and 0.67 (95% CI, 0.59–0.74), respectively. The cutoff value of 60 pg/mL for T3 levels displayed a sensitivity of 81% and specificity of 64% for predicting death, with an OR of 4.89 (95% CI, 2.48–9.66).

The evolution to CCI was more frequent in patients with NTIS than in those with normal TH levels (62% vs. 23.5%, *p* < 0.001). Only SAPS III and T3 levels independently predicted the evolution to CCI (Table 2). Lower levels of T3 yielded an AUC of 0.75 (Figure 3B). The cutoff value of 60 pg/mL displayed a sensitivity of 72.5% and specificity of 72% for predicting the evolution to CCI, with an OR of 2.20 (95% CI, 1.61–3.01).

## 3. Discussion

This prospective clinical study explored the independent association among low T3 levels, high expression of DIO3 in immune cells, and adverse outcomes in patients with sepsis and septic shock who were admitted to the ICU. We observed a high expression of DIO3 in immune cells, which was related to disease severity and outcomes. Patients with low T3 levels at ICU admission were at a higher risk of progression to CCI and mortality within 28 days. The T3 levels alone were independently associated with worse outcome, without covariance with other commonly used severity scores. T3 levels <60 ng/dL displayed the best sensitivity and specificity to predict mortality and the evolution to CCI.

The prevalence of low T3 syndrome in ICU settings varies substantially, ranging from 16% to 80% [10,11,29,30,31,32]. The prevalence of low T3 levels in 86.5% of the patients was partially explained by the cohort being exclusively composed of patients with sepsis. Sepsis is characterized by the simultaneous release of proinflammatory and anti-inflammatory mediators, with the intensity of the response depending on multiple factors, including the host and the associated pathogen. The resulting proinflammatory cytokines cause the activation and proliferation of leukocytes and elicit a complete immune response [33].

The interaction between the complex network of immunity and TH function plays a role in lowering T3 levels in sepsis, despite the difficulty in identifying a simplistic model [34,35,36]. One of the possible paths involves the altered expression of deiodinase genes in the immune cells due to oxidative stress. However, this has not been studied yet. In patients with sepsis, the levels of sulfhydryl and T3 increased in parallel in both patient groups, thereby suggesting the consumption of thiol products and the oxidation of protein-bound sulfhydryl groups may alter thyroid hormone metabolism in these patients. Moreover, we observed an inverse correlation between protein oxidation (carbonyl levels) and T3 levels, which reinforced the role of oxidative stress in the system. The high levels of protein oxidation directly affect the thiol regenerating system, by modulating GSH and NADPH levels, in such patients [22]. Here, we demonstrated the induction of DIO3 in immune cells, which further decreased plasma T3 levels and increased the inactivation of T4 to rT3. Recently, we reported on a correlation between sulfhydryl and GSH levels with augmented DIO3 activity in several tissues under NTIS [23]. The present study showed induced DIO3 in white cells, paralleled with increased oxidative stress and diminished thiol levels. The sulfhydryl correlation with DIO3 expression in blood favors the idea that glutathione regulates or is one of the regulators of DIO3 expression. These findings, among others published through the years, add to our understanding of the mechanism by which the administration of the antioxidant NAC, which sustains the redox state of cysteine protein residues, restores redox equilibrium and prevents the deregulation of TH concentrations in humans [37]. We and others, just to highlight a few examples, have extensively shown that the causes/consequences of low T3 syndrome can be detected in all organs of the mammalian body [8,21,23,28,38]. However, a key issue is that we cannot perform muscle or liver biopsies on our patients to determine if they have low T3 syndrome or if type 3 deiodinase expression is altered in multiple tissues. Moreover, several studies already addressed this aspect. The main objective of our study was to determine the presence and prognostic role of type 3 deiodinases on an easily obtainable tissue (blood) from patients who were critically ill but still alive.

Some limitations of the study are noted: (1) the outcome data were limited to in-hospital events; (2) we did not measure rT3 levels, preventing the complete characterization of NTIS; and (3) we did not measure DIO3 expression or activity in separated neutrophils or macrophages but only measured DIO3 mRNA in the whole cell pool, which could have underestimated the change in DIO3 expression in one of these lineages.

Our findings have clinical and mechanistic relevance since low T3 concentrations were associated with poor outcomes at ICU admission, thereby independently predicting progression to CCI and death. Moreover, this study adds to our current understanding of how the induced expression of DIO3 in immune defense cells plays an important role in lowering circulating T3 levels. Patients with lower T3 levels displayed a significantly higher risk of persistent organ dysfunction and mortality. Our observations provide a unified hypothesis to explain the low T3 plus high DIO3 expression in immune cells of these patients and is a major step toward unraveling this long-standing enigma, thus helping us identify a previously unrecognized combinatorial pathway. These results represent an integrative measure of multiple harmful pathological processes in patients with critical illnesses, such as inflammation and energetic imbalance, which are associated with adverse outcomes. Finally, it will be important to investigate if antioxidants, such as NAC, could be beneficial as an adjuvant therapy with other therapeutic measures in critically ill patients.

## 4. Materials and Methods

### 4.1. Patients

**Figure 4:**
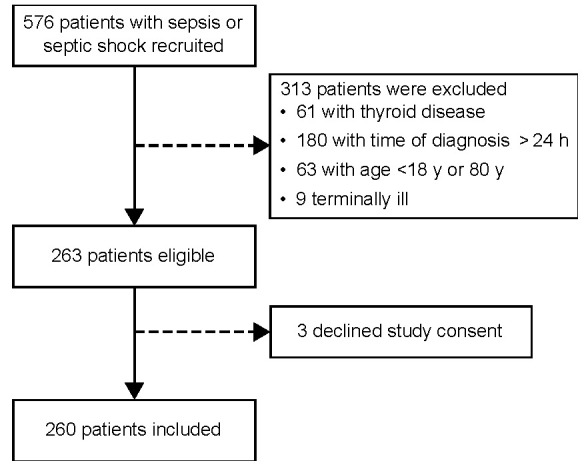
Flowchart showing patient selection.

This prospective cohort study was performed between October 2017 and April 2019 and recruited 576 consecutive adults admitted to the ICU of a tertiary hospital in southern Brazil. Patients diagnosed with sepsis or septic shock within 24 h of evolution were eligible for the study. Sepsis was defined as organ dysfunction (an increase in the SOFA score by ≥2 points) caused by a dysregulated host response to infection. Septic shock was defined as a subset of sepsis characterized by profound circulatory, cellular, and metabolic abnormalities, clinically identified by vasopressor requirement to maintain a mean arterial pressure ≥65 mmHg, and serum lactate level >2 mmol/L (>18 mg/dL) in the absence of hypovolemia [39]. Low T3 referred to levels under the normal range, concomitant with normal/low-normal TSH serum levels [40]. The exclusion criteria were as follows: (1) age <18 years or >80 years; (2) a history of primary thyroid disease; (3) pregnancy or immediate postpartum period; and/or (4) imminent death or the directive of exclusive palliative care [30,32]. Based on the prevalence of NTIS and local mortality for patients with septic shock, we calculated the sample size to provide a statistical power of 90% for determining an absolute difference of 20% in the mortality between the two groups (assuming a two-sided level) using Epitools Epidemiological Calculators [41]. Accordingly, 260 patients were enrolled (Figure 4).

### 4.2. Blood Measurements

Venous blood samples were obtained within the first 24 h. We collected data related to the severity score systems, namely, the Charlson Comorbidity Index, SAPS III, and SOFA, on the first day following ICU admission. THs were measured by electrochemiluminescent immunoassay (ADVIA Centaur XP; Siemens, Munich, Germany). Its normal ranges were as follows: free T4 (fT4), 0.93–1.7 ng/dL; T4, 4.6–12 ng/dL; T3, 75–200 ng/dL; and TSH, 0.27–4.2 mU/L. We did not study FT3 because the cutoff point for low T3 syndrome is not fully established for FT3. Moreover, T3 analysis is widely available even in small clinical centers and laboratories. Finally, FT3 has a wider range of variability, and it is not reliably validated among different centers. Additionally, the sulfhydryl content was determined as described by Aksenov and Markesbery (2001) [42], using the reduction in 5,5′-dithiobis(2-nitrobenzoic acid) (DTNB) by thiols. DTNB absorbance was measured using a spectrophotometer at 412 nm. Results were expressed as nmol TNB/mg protein. Carbonyl content was measured according to Zannata et al. [43]. The difference between samples treated with 2,4-dinitrophenylhydrazine and those treated with HCl (blank) was used to calculate the carbonyl content. The carbonyl content was determined at 370 nm and calculated using the millimolar absorption coefficient of hydrazine (e370 nm = 21 M^−1^ cm^−1^); the results were expressed in nmol carbonyl/mg of protein.

### 4.3. Real-Time Quantitative Polymerase Chain Reaction (PCR)

For reverse transcription of 250 ng of RNA into cDNA, we used the SuperScript VILO Master Mix IV (Thermo Fisher Scientific, Waltham, MA, USA), following the manufacturer’s protocol. The cDNA was then amplified by quantitative real-time PCR (qPCR). The qPCR experiments were performed by monitoring, in real time, the increase in fluorescence of the SYBR^®^ Green dye [44]. Primers for *DIO3* were 5′-TCCAGAGCCAGCACATCCT-3′ and 5′-ACGTCGCGCTGGTACTTAGTG-3′. Primers for reference genes were beta-actin (*ACTB*) 5′-ACAGCCTGGATAGCAACGTACA-3′ and 5′-AGGCCAACCGCGAGAAG-3′ and beta2-microglobulin (*β2M*) 5′-ACAAGTCTGAATGCTCCACT-3′ and 5′-CTATCCAGCGTACTCCCAAG-3′. Primers were designed using published human gene sequences in the Primer Express 3.0 Software (Thermo Fisher Scientific, Waltham, MA, USA). PCR reactions were performed using 5 μL of 1X Fast SYBR Green Master Mix (Thermo Fisher Scientific, Waltham, MA, USA), 0.5 μL (1 ng/μL) of forward and reverse primers for the gene, and 1 μL of cDNA (12.5 ng/μL for DIO3), in a total volume of 10 μL. Then, the cDNA was amplified by qPCR in the ViiA7 Real-Time PCR System (Thermo Fisher Scientific, Waltham, MA, USA). The qPCR specificity was determined using melting curve analyses. Each sample was assayed in triplicate, and a negative control was included in each experiment. Quantification was performed using the comparative ΔΔCq method. The ΔΔCq method calculates changes in gene expression as relative fold differences (n-fold changes) between an experimental and an external calibrator sample [45].

### 4.4. Statistical Analysis

Categorical data are presented as frequencies and were analyzed using the χ^2^ test or Fisher’s exact test. Quantitative data with normal distribution are presented as mean ± SD and were analyzed using the two-way ANOVA test. Non-parametric variables are presented as median ± interquartile range and were analyzed by the Mann–Whitney U test. We determined the correlations between variables by Spearman’s correlation test. We performed a multivariate binary logistic regression by the independent mortality predictor’s assessment. Moreover, we included associated factors (*p* < 0.05 was the retention criterion for each factor) from the univariate analysis in the multivariable logistic regression analysis. The power of the association between risk factors and outcomes was expressed as the odds ratio (OR). We applied the receiver operating characteristic (ROC) curve to set the critical point for predicting the mortality for T3 levels. The results are presented as the area under the curve (AUC) with sensitivity and specificity. Data were analyzed using the Statistical Package for Social Sciences (SPSS) version 23 software (SPSS Inc., Chicago, IL, USA), with a significance of *p* < 0.05.

## 5. Conclusions

The present study provided evidence for low T3 levels as an independent prognostic factor in patients with sepsis and septic shock, with reduced T3 levels at ICU admission being associated with a higher risk of progression to CCI and death. Furthermore, the high expression of DIO3 in circulating white cells is a novel mechanism that further explains the reduction in the T3 levels in patients with sepsis.

## Figures and Tables

**Table 1:** Baseline characteristics and blood concentration of biomarkers in all patients and a comparison by the diagnosis at admission to the ICU and outcomes.

	All Patients (*n* = 260)	Sepsis (*n* = 88)		*p*-Value	Septic Shock (*n* = 172)		*p*-Value
		Survivors (*n* = 75)	Non-Survivors (*n* = 13)		Survivors (*n* = 91)	Non-Survivors (*n* = 81)	
Demographic characteristics							
Age, years	58.3 ± 14.7	56.8 ± 14.2	56.9 ± 21.3	0.99	56 ± 15.1	63.4 ± 11.8	0.001
Male sex, %	55.4	69.2	57.3	0.31	51.6	55.6	0.36
Site of infection, %							
Pulmonary	41.1	43.2	38.6	0.01	37.8	43.2	0.5
Abdominal	26.7	17.6	46.2		28.9	29.6	
Urinary tract	10.9	13.5	0		12.2	8.6	
Others	21.3	25.7	15.4		21.1	18.5	
Healthcare-associated infection (%)	42.7	40.0	76.9	0.01	42.9	39.5	0.38
Predictive scoring systems on admission							
SAPS	71.8 ± 17.7	62.7 ± 14.2	68.1 ± 14.2	0.22	73 ± 16.8	81.8 ± 18.8	0.003
SOFA	7.8 ± 3.4	5.0 ± 2.6	6.0 ± 3.2	0.24	9.0 ± 2.6	10.0 ± 3.1	0.015
CHARLSON	4.5 ± 2.2	3.9 ± 2.3	5.1 ± 1.9	0.11	4.3 ± 2.2	5.6 ±1.9	<0.001
Routine laboratory findings							
Lactate (<2.2 mol/L)	2.9 ± 2.4	1.8 ± 1.3	2.1 ± 1.6	0.50	2.9 ± 2.1	4.8 ± 3.4	<0.001
C-reactive protein (mg/L)	201.8 ± 121	183.1 ± 133	216.9 ± 120	0.41	195 ± 109	224 ± 126	0.12
White blood count (×10)	13.6 (8–18.6)	16.3 (11.5–19.7)	13.1 (7.9–16.7)	0.07	15.2 (8.9–23.1)	11.4 (4.7–17.7)	0.8
Thyroid hormones							
NTIS (%)	86.5	70.7	100	0.01	89.8	98.9	0.02
TSH (0.27–4.2 UI/mL)	2.26 ± 2.34	2.1 ± 2.1	2.3 ± 1.8	0.74	2.4 ± 2.4	2.1 ± 2.4	0.44
T4 (4.6–12 ng/dL)	5.4 ± 2.1	6.5 ± 2.1	4.6 ± 0.8	0.001	5.3 ± 1.9	4.6 ± 2.1	0.018
fT4 (0.93–1.7 ng/dL)	1.05 ± 0.35	1.17 ± 0.33	1.01 ± 0.36	0.10	1.05 ± 0.3	0.95 ± 0.4	0.06
T3 (75–200 ng/dL)	56.2 ± 16.9	68.1 ± 17.3	49.7 ± 8.8	<0.001	57.2 ± 16.4	46.6 ± 12.5	<0.001
Outcomes							
LOS, days	4 (3–10)	4 (2–6)	5 (3–12)	0.98	6 (3–14)	4 (2–9)	0.002

Abbreviations: fT4: free T4; ICU: intensive care unit; LOS: length of stay; NTIS: non-thyroidal illness syndrome; SAPS: Simplified Acute Physiology Score; SOFA: Sequential Organ Failure Assessment score; T3: triiodothyronine; T4: thyroxine; TSH: thyrotropin. Categorical data are presented as frequencies and were analyzed using the χ2 test or Fisher’s exact test. Quantitative data with normal distribution are presented as mean ± SD and were analyzed using the Student’s t-test. Non-parametric variables are presented as median ± interquartile range and were analyzed using the Mann–Whitney U test. A *p* < 0.05 is considered statistically significant. Correlation between T3 and oxidative parameters.

**Table 2:** Predictors of death within 28 days as identified by univariate analysis and multivariate logistic regression.

Univariate Analysis					
		Survivors (*n* = 166)		Non-Survivors (*n* = 94)	*p*-Value
Age, years		56.3 ± 14.6		62.5 ± 13.5	0.001
SAPS		68.3 ± 16.5		79.3 ± 18.7	<0.001
SOFA		7.2 ± 3.3		9.4 ± 3.3	<0.001
CHARLSON		4.1 ± 2.2		5.4 ± 1.9	<0.001
Lactate (<2.2)		2.4 ± 1.9		4.4 ± 3.3	<0.001
C-reactive protein		190 ± 120		223 ± 125	0.04
T4 (4.6–12 ng/dL)		5.86 ± 2.1		4.55 ± 2.01	<0.001
fT4 (0.93–1.7 ng/dL)		1.12 ± 0.34		0.96 ± 0.37	0.001
T3 (75–200 ng/dL)		62.09 ± 17.64		47.05 ± 12.1	<0.001
**Multivariate Analysis**					
	**Estimate**	**SE**	**Odds Ratio**	**95% CI**	***p*** **-Value**
Intercept	−0.849	0.4903	0.428	0.164–1.118	0.083
T3	−0.038	0.0051	0.962	0.953–0.972	0.0001
T3 < 60 ng/dL	1.58	0.34	4.89	2.48–9.66	0.0001
SAPS III	0.012	0.0052	1.012	1.002–1.023	0.017
Charlson	0.149	0.0411	1.161	1.071–1.256	0.0001

Abbreviations: fT4: free T4; SAPS: Simplified Acute Physiology Score; SOFA: Sequential Organ Failure Assessment score; T3: triiodothyronine; T4: thyroxine.

## Data Availability

The data presented in this study are available on request from the corresponding author. The data are not publicly available due to ethical restrictions.
